# The Impact of Mode of Delivery and Postpartum Conditions on Breastfeeding: A Cross-Sectional Study

**DOI:** 10.3390/healthcare12020248

**Published:** 2024-01-18

**Authors:** Anita Hulman, Annamária Pakai, Tímea Csákvári, Viola Keczeli, Katalin Varga

**Affiliations:** 1 Doctoral School of Health Sciences, Faculty of Health Sciences, University of Pécs, 4 Vörösmarty Str., H-7621 Pécs, Hungary; 2 Institute of Basics of Health Sciences, Midwifery and Health Visiting, Faculty of Health Sciences, University of Pécs, 4 Vörösmarty Str., H-7621 Pécs, Hungary; 3 Department of Health Economics and Health Care Management, Institute of Health Insurance, Faculty of Health Sciences, University of Pécs, 33 Landorhegyi Str., H-8900 Zalaegerszeg, Hungary; 4 Department of Affective Psychology, Institute of Psychology, ELTE Eötvös Loránd University, 46 Izabella Str., H-1064 Budapest, Hungary

**Keywords:** caesarean section, skin-to-skin contact, rooming-in, breastfeeding, pacifier utilization, nurse, midwife, support

## Abstract

Breast milk is the optimal and essential source of nutrients for babies. Many women, however, do not breastfeed or stop early after giving birth, often due to lack of support. For newborns delivered by caesarean section, there is often a delay, or no skin-to-skin contact after birth; thus, early breastfeeding is not achieved. Separation, complementary feeding or pacifier use also limits the mother’s ability to breastfeed. A quantitative, cross-sectional study was conducted. Sociodemographic data, the mode of delivery and postpartum circumstances, information on breastfeeding, and the method and duration of feeding were collected (n = 2008). Two-thirds of children born by caesarean section did not have skin-to-skin contact after birth (*p* < 0.001). Lack of rooming-in placement increased the incidence of more frequent complementary feeding (*p* < 0.001) and shortened the duration of exclusive breastfeeding (*p* < 0.001). The duration of breastfeeding may also be negatively affected by scheduled feeding (*p* = 0.007) and pacifier utilization (*p* < 0.001). The mode of delivery and postpartum circumstances directly affecting the mother and the newborn can affect the feasibility of breastfeeding and the duration of exclusive and partial breastfeeding. For positive breastfeeding outcomes, skin-to-skin contact immediately after birth, rooming-in and unrestricted, demand breastfeeding, as well as the avoidance of the use of pacifiers, are recommended.

## 1. Introduction

Breast milk is the easiest food for babies to digest, and the best way for them to access the nutrients they need to grow and develop. Breast milk also provides the child with antibodies and other protective substances that increase disease resistance. At the same time, breastfeeding provides not only nutrition but also immunological protection and safety for the baby [1]. Exclusive breastfeeding is also cost-effective and a healthy behavior for the mother–child dyad [2]. Nevertheless, the rate of exclusive breastfeeding is insufficient [3].

Recognizing the impact of hospital conditions and the postnatal day on breastfeeding, the WHO and UNICEF (United Nations Children’s Emergency Fund) established the Baby-Friendly Hospital Initiative in 1991, with the main aim of creating maternity facilities that support breastfeeding as the norm by introducing the “Ten Steps to Breastfeeding Success”, to ensure newborn babies get the best start in life. It emphasizes the need for skin-to-skin contact immediately after birth and for rooming-in of the newborn and mother 24 h after birth and throughout the hospital stay. It also recommends unrestricted, demand breastfeeding and the avoidance of the use of pacifiers. It further stresses that substitute fluids (tea, sugar solution, formula, etc.) should only be given for medical reasons and on medical advice [4]. In a study in Kilimanjaro, most mothers gave colostrum to their babies; only a small proportion (6.4%) gave pre-lacteal foods. Giving colostrum and not pre-lacteal food was significantly associated with breastfeeding beginning within one hour after birth [5].

The WHO also recommends that optimal feeding includes skin-to-skin contact after birth, the initiation of early breastfeeding within 1 h of birth, guided by the newborn, exclusive breastfeeding for the first 6 months of the child’s life, and then breastfeeding with complementary feeding until 2 years of age or beyond [6].

Wu et al. report that 24-h rooming-in placement is associated with the exclusive breastfeeding at 1 and 3 months postpartum [7]. The WHO and UNICEF recommend that mothers and newborns should have skin-to-skin contact immediately after vaginal delivery and as soon as the mother is awake and responsive following a caesarean section [8]. Moore et al. have also found that skin-to-skin contact immediately after birth and lasting at least 1, preferably up to 2 h has numerous physiological and psychological effects [9]. It prevents separation distress, calms both the mother and the newborn, increases maternal sensitivity, and results in improved suckling patterns and breastfeeding indicators. Sandhi et al. reported that nearly two-thirds of Indonesian mothers exclusively breastfed their babies up to 6 months of age. Exclusive breastfeeding rates were found to be higher for those infants at 6 months of age, who had skin-to-skin contact with their mothers within 1 hour of birth [10].

Moore et al. and Boakye-Yiadom et al. state that early skin-to-skin contact increases the duration of breastfeeding after normal vaginal delivery [9,11] Hauck et al. found that women who gave birth by caesarean section were less likely to initiate breastfeeding, which was also supported by the research of Tahsina et al. [12,13]. Caesarean section reduces the initiation of breastfeeding, delays the time to first breastfeeding and reduces the chance to breastfeed exclusively, but also significantly delays the onset of lactation and increases the likelihood of complementary feeding [14]. Akylidiz et al. report that obstetric interventions adversely affect breastfeeding and that one out of two newborns do not have skin-to-skin contact with their mothers immediately after birth. According to their study, newborns were most often breastfed for the first time by their mothers half an hour after the hospital protocol had been completed [15]. Cadwell reported higher rates of early breastfeeding among mothers in rooming-in. The nearly two-thirds exclusive breastfeeding rate observed in non-separated mother–infant pairs may be halved in the case of separation. Even brief separation has been shown to have adverse effects [16]. Kopcsó et al. found a positive association between exclusive and demand breastfeeding. In their study, more than half of the infants were breastfed until 6 months of age, and a quarter were exclusively breastfed until the same age [17]. In their meta-analysis, Karabulut et al. describe a positive association between pacifier use and a shorter duration of exclusive and partial breastfeeding [18].

Buccini et al. found an association between pacifier utilization and cessation of exclusive breastfeeding. Reducing pacifier use increased exclusive breastfeeding rates by 5.5% among infants younger than 6 months of age [19]. In addition, Deus et al. reported that the duration of pacifier use negatively correlated with breastfeeding duration and the incidence of anterior open biting [20].

This study aimed to assess the relationship between the mode of delivery, postpartum conditions, and the development of breastfeeding among Hungarian mothers.

## 2. Materials and Methods

### 2.1. Study Design and Objective

Our quantitative, cross-sectional study’s objective was to assess the characteristics of infant feeding in the perinatal and postpartum settings to improve the duration and exclusiveness of breastfeeding by exploring possible correlations.

### 2.2. Sample Characteristics

A non-random, targeted expert sampling method was used. The survey was conducted online using a self-completion questionnaire between 26 March 2021 and 18 July 2021. The questionnaire was made publicly available on social platforms for mothers and gravidas. Participation was anonymous and voluntary among Hungarian mothers living either in Hungary or abroad. The eligibility criteria included biological motherhood, having at least one living child born after the 37th week of gestation and raised in the mother’s care at the time of completing the questionnaire. Exclusion criteria were non-biological motherhood, being gravid with their first child, and not completing the compulsory questions correctly. Other exclusion criteria included a report of a congenital and/or acquired physical or mental illness of the child or mother which made breastfeeding impracticable. We only asked mothers about their most recent birth; however, if the mother had more than one biological child under the age of 60 months, she could fill in the questionnaire for them. A total of 2505 people responded to the questionnaire, of whom 2008 were further analyzed after exclusion.

### 2.3. Data Collection

Prior to the research, a comprehensive systematic literature search was conducted in academic search systems and publishers (Pubmed, Medline, Google Scholar, Wiley, Scopus, BMC). Relevant literature was categorized and further organized according to logical and content criteria. Subsequently, a pilot study involving six participants was conducted to correct any errors based on the comments received. For our research, data were collected through a self-completed questionnaire survey. The online data collection lasted for 4 months and was mainly motivated by the different localization and the targeting of a high number of items. The questionnaire consisted of 74 questions, with closed and semi-open questions, thus providing the opportunity to form an independent opinion.

The questionnaire consisted of questions about sociodemographic factors, followed by questions on anthropometric data. We then asked the mothers in detail about the mode and events of delivery, as well as about the circumstances of the days spent in the hospital, such as the establishment of skin-to-skin contact between mother and newborn, the type of accommodation, as well as the timing and length of breastfeeding.

Several steps were taken to ensure valid participant data. The questionnaire was published on a community platform for pregnant and postpartum mothers, and it was only available to registered users, where moderators try to prevent invalid profiles. In addition, the questionnaire contained several semi-open questions which can only be answered by real people. Several of the questions were compulsory, i.e., it was not possible to submit the questionnaire without answering them. In addition, we believe that since the questionnaire contained 74 questions, the time taken to complete it would have been about 20 min, also making it difficult for robots to complete it. Furthermore, during the analyses, we excluded responses from participants who answered the question irrelevantly.

### 2.4. Statistical Analysis

Statistical analysis was performed using Microsoft Excel 365 and SPSS 25.0 software. Descriptive statistics included absolute and relative frequencies, mean, standard deviation, minimum and maximum values. An χ^2^ test, an ANOVA and a two-samples *t*-test were used to analyze the relationship and differences between the variables under study. The results were presented as frequencies, and the significance level was set at *p* < 0.05 [21].

The study was conducted in accordance with the Declaration of Helsinki and approved by the Ethics Committee of the Markusovszky Hospital in Szombathely (13/2021).

## 3. Results

We included 2008 mothers in the study. The mean age of mothers at delivery was 31.04 years (SD = 4.93, min = 17, max = 50). In terms of educational attainment, 61.9% (n = 1242) had college or university degrees, 31.9% (n = 640) had completed high school, another 4.8% (n = 97) had completed vocational school, while 1.4% (n = 29) had completed 8 years of primary school or less.

Most respondents (73.3%, n = 1472) answered the questions for their first child, 20.4% (n = 409) for their second child, 4.4% (n = 88) for their third child and 2.6% (n = 38) for their fourth (or more) child.

For breastfed infants (n = 1659), the mean exclusive breastfeeding duration was 4.97 months (SD = 2.24, min = 0, max = 24). Among the infants, 67% (n = 1346) were exclusively breastfed for the first 6 months of their lives. The duration of exclusive breastfeeding was positively correlated with the duration of breastfeeding (r(1643) = 0.383, *p* < 0.001). Mean breastfeeding duration was 10.73 months (SD = 8.36, min = 0, max = 60).

A significant difference was found between the timeliness of breastfeeding and the duration of breastfeeding (t(1718) = 6.44, *p* < 0.001). Mothers who breastfed their babies on demand did so for more than 12 months (M = 12.22, SD = 8.39, min = 0, max = 60), while mothers who breastfed on a schedule breastfed for 8 months (M = 8.11, SD = 5.68, min = 0, max = 31).

### 3.1. Possible Effects of the Mode of Delivery on Skin-to-Skin Contact, Formula Feeding and Pacifier Use

There was a remarkable difference in the extent of skin-to-skin contact after childbirth between the different types of delivery. The highest rate of skin-to-skin contact, more than 90%, was among parents who gave birth vaginally, while less than 30% of mothers who had an emergency caesarean section had skin-to-skin contact immediately after delivery (Table 1).

There was also a significant difference in the time of mother-infant skin-to-skin contact according to the mode of birth. Of those who gave birth vaginally, four-fifths had their baby immediately, while nearly a quarter of those who had an elective caesarean section had their baby within 30 min or a similar proportion had their baby more than 2 h later. The highest proportion (one-third) of mothers who had their baby after birth were those who had an emergency caesarean section; these were also the latest to have their baby (more than 2 h) (Table 1).

However, there was also a significant difference in formula feeding between children born virginally and those born by caesarean section. Children born by emergency caesarean section had the highest proportion: almost two-thirds of children who were formula-fed at once in their life, while children born vaginally had the highest proportion (almost 50% who were never formula-fed) (Table 1).

There was a significant difference in pacifier utilization between babies born by different means. The highest prevalence was found in babies born by emergency caesarean section, with over 60%, while the highest rates of non-use were found in babies born vaginally, with slightly less than 50% (Table 1).

### 3.2. Skin-to-Skin Contact in Breastfeeding

We found a significant difference in the supplementary formula feeding of newborns with and without skin-to-skin contact immediately after birth (χ^2^(df) = 111.906(1), *p* < 0.001). In 62.1% (n = 830) of the newborns with skin-to-skin contact, no complementary feeding was given, whereas, in a similar proportion of 64.5% (n = 359) of the non-skin-to-skin contact infants, complementary feeding was given during the hospital stay (Table 2).

A significant difference was found between the time of skin-to-skin contact and the length of exclusive breastfeeding (t(1638), *p* = 0.003). Infants who were placed on their mother’s chest within 30 min were exclusively breastfed for 5.05 months (SD = 2.16, min = 0, max = 24), while those who were in skin-to-skin contact with their mother after more than 30 min were exclusively breastfed for 4.64 months (SD = 2.49, min = 0, max = 20) (Table 3).

Furthermore, a significant difference was found between the onset of skin-to-skin contact and the length of partial breast milk feeding (t(1913), *p* = 0.021). Newborns who had skin-to-skin contact within 2 h after birth were breastfed for 10.95 months (SD = 8.42, min = 0, max = 60), while those who started skin-to-skin contact with their mothers more than 2 h later were breastfed for 9.67 months (SD = 8.05, min = 0, max = 59), i.e., for at least half a month less (Table 3).

### 3.3. Relationship between the Method of Placement and Infant Feeding

We found a significant association between the mode of delivery and rooming-in placement. The highest rates of rooming-in were among parents who delivered vaginally and the lowest among those having an emergency caesarean section.

Furthermore, there was a significant difference between postpartum skin-to-skin contact and rooming-in. Nearly half of mothers in non-rooming-in placements had their babies more than 2 h after delivery, while three-quarters of mothers in rooming-in placements had their babies immediately after delivery.

We also found a significant relationship between rooming-in and the provision of complementary feeding to the newborn during hospitalization. More than half of the newborns in rooming-in did not receive any form of complementary feeding, compared with nearly four-fifth of the newborns separated from their mothers (Table 4).

Nevertheless, a positive correlation was shown between rooming-in and the regularity of breastfeeding. Although a significant majority of mothers not placed in rooming-in at the hospital also demanded to breastfeed their babies, the proportion was found to be almost 10% higher for mothers placed with their newborns (Table 4).

Mean breastfeeding periods for infants co-located at the time of hospitalization was 10.80 months (SD = 8.32, min = 0, max = 60), while it was 9.6 months in infants not co-housed who were breastfed (SD = 8.57, min = 0, max = 59), with no significant difference between the two groups in the length of breastfeeding (t(1931) = −1.52, *p* = 0.128) (Table 4).

### 3.4. Relationship between Pacifier Use and Breastfeeding

There is also a significant difference in breastfeeding between infants who use a pacifier and those who do not use one for the first 6 months after birth. χ^2^(1) = 202.775, *p* ≤ 0.001). Among infants who were breastfed for the first 6 months, a majority of 58.8% (n = 682) did not use a pacifier, while among those who were not exclusively breastfed, 73.2% (n = 621) used one.

Infants who used pacifiers differed significantly from those who did not use pacifiers in the duration of exclusive breastfeeding (t(1657) = 10.625, *p* < 0.001). Infants who used pacifiers were exclusively breastfed for 4.38 months (SD = 2.47, min = 0, max = 24), compared to 5.51 months (SD = 1.86, min = 0, max = 17) for infants who did not use one. Infants who never used a pacifier were exclusively breastfed for longer periods (Table 5).

In addition, partial breastfeeding also lasted (approximately 5 months) longer among children who did not use a pacifier. Breastfeeding lasted 13.6 months (SD = 8.82, min = 0, max = 59) for children who did not use a pacifier and 8.28 months (SD = 7.09, min = 0, max = 60) for children who did use a pacifier; this difference is significant (t(1940) = 14.728, *p* < 0.001).

## 4. Discussion

Our results are in accordance with several previous studies [9,12,14,15]. In the case of infants born via caesarean section, there is a lower and delayed occurrence of skin-to-skin contact with the mother, a factor known to affect breastfeeding indicators. According to our research, skin-to-skin contact between the mother and newborn occurred most frequently during the golden hour (within 2 h after birth) in natural (vaginal) deliveries, but least frequently in caesarean section deliveries. Akylidiz et al. describe in their study how immediately after birth, only every second newborn had skin-to-skin contact with the mother [15]. In our study, we obtained similar results, with just over two-thirds of newborns establishing skin-to-skin contact with their mothers after birth.

Mothers who delivered via caesarean section tended to initiate breastfeeding later [9,12,13,14]. Our study confirmed this, showing that four-fifths of vaginally delivering mothers breastfed their child immediately, while this rate did not even reach 10% for planned caesarean section births or emergency caesarean section births. Similar to Stevens et al. [14], our research also states that caesarean section negatively affects breastfeeding initiation. Furthermore, infants who had skin-to-skin contact with their mothers after birth were significantly less likely to receive supplementary formula feeding compared to those who did not have immediate contact.

The World Health Organization emphasizes the importance of immediate skin-to-skin contact after birth, starting breastfeeding within the first hour, and continuing exclusively for the first 6 months, followed by appropriate complementary feeding up to 2 or more years [6]. Our survey found that skin-to-skin contact within the first hour, as recommended by the WHO and UNICEF, was achieved in two-thirds of mothers, who exclusively breastfed their infants up to 6 months [4,6].

The duration of skin-to-skin contact is also an important factor. Similar to other research results, indicating a higher exclusive breastfeeding rate for newborns breastfed within the first hour, we found that breastfeeding lasted significantly longer for infants who had skin-to-skin contact with their mothers within the first 30 min after birth.

Skin-to-skin contact extends the duration of breastfeeding [9,11], a finding consistent with our results. The duration of breastfeeding was longer for infants breastfed within the first 2 hours of skin-to-skin contact.

In addition, infants with skin-to-skin contact were less likely to receive formula supplementation than those with skin-to-skin contact after 2 h. However, the mean duration of breastfeeding did not extend up to 1 year for the infants in our survey, and thus, the recommended duration by the WHO [6] of at least 2 years was not achieved.

The significance of the rooming-in system is supported not only by the WHO [2,22], but also by several studies [16,23]. Cadwell et al. mention in their study that rooming-in results in a higher rate of early breastfeeding, and not rooming-in negatively affects the duration of breastfeeding. Our study also supported their findings, showing that mothers in the rooming-in system had a shorter separation time compared to those in the non-rooming-in system. Nearly half of those not in the rooming-in system received their child more than 2 h after birth, whereas more than half in the rooming-in system received their child immediately after delivery.

Cadwell also reported a decreasing exclusive breastfeeding rate in non-rooming-in placement, a finding supported by our results. Infants who spent more than 24 h in continuous contact with their mothers were less likely to receive formula supplementation, while the majority of infants separated from their mothers received formula [16]. The negative impact of separation on breastfeeding progression was also evident in our results. Infants placed in the rooming-in system were breastfed for 1 month longer than those in the non-rooming-in system, although the difference was not significant in the duration of exclusive breastfeeding between the two groups.

The WHO recommends on-demand breastfeeding [4,8], and the majority of the participating mothers in our study adhered to this recommendation. While mothers in the rooming-in system breastfed their infants more responsively, there was no significant difference compared to mothers with infants placed separately. Kopcsó et al. pointed out the relationship between responsiveness and the duration of exclusive breastfeeding [17], also supported by our results. Infants exclusively breastfed were breastfed 4 months longer.

The use of pacifiers was associated with a decrease in the duration of exclusive breastfeeding [19]. The decrease in pacifier use was related to an improvement in the rate of exclusive breastfeeding. A correlation between pacifier use and breastfeeding duration was found by other studies as well [18,20]. We obtained similar results, with exclusive breastfeeding lasting significantly longer in children who did not use pacifiers. Additionally, children exclusively breastfed for the first 6 months were significantly less likely to use pacifiers. In our study, pacifier use was most common in those born via emergency caesarean section, followed by those born via planned caesarean section, and least common in infants born vaginally. Exclusive breastfeeding occurred significantly longer for infants not using pacifiers.

Finally, in terms of our research, the most comprehensive international guideline is the “Ten Steps to Successful Breastfeeding” [4], which recommends immediate skin-to-skin contact after delivery, co-placement with the mother during hospital care and on-demand feeding. While most mothers fulfilled these recommendations, adherence to the recommendation of avoiding pacifiers was not as common.

One conclusion of the study is that the protocols for supplementary feeding, which proved to be a factor influencing breastfeeding, vary between obstetric institutions.

A limitation of our study is that our sample is not representative in any way, and the fact that the survey was limited to mothers with internet access may be a source of bias. All our data are self-reported and therefore undetected errors or biases may occur.

Another limitation could be the retrospective method of data collection, and the possible recall bias. To attempt to eliminate recall bias, the child for whom the mother completed the questionnaire must be no more than 60 months (5 years) old.

## 5. Conclusions

The successful implementation of breastfeeding is helped by the first onset of skin-to-skin contact within 2 h after birth (golden hours) and the 24-h co-placement of the mother and the newborn (rooming-in), which occur earliest and most frequently in vaginal deliveries. In addition, on-demand breastfeeding and the avoidance of pacifier use also promote the duration of (exclusive) breastfeeding.

The mode of delivery and the circumstances of the postpartum period are significantly associated with positive breastfeeding outcomes. To improve our obstetric and breastfeeding indicators, it is key to be aware of and apply the latest evidence-based research and recommendations.

## Figures and Tables

**Table 1:** Postpartum care and infant feeding based on the mode of delivery.

	Vaginal Birth (n (%))	Elective c-Section (n (%))	Emergency c-Section (n (%))		*χ^2^*(2)	*p*
Skin-to-skin contact						
Present	1157 (91.2%)	96 (38.7%)	131 (27.3%)	793.425		<0.001
Absent	111 (8.8%)	152 (61.3%)	348 (72.7%)			
Time of placing the newborn on the mother’s chest						
Immediately	1027 (80.8%)	20 (8.2%)	12 (2.6%)	1300.787		<0.001
After 10 min	144 (11.3%)	24 (9.8%)	39 (8.4%)			
After 30 min	21 (1.7%)	59 (24.2%)	95 (20.4%)			
After 1 h	24 (1.9%)	47 (19.3%)	100 (21.5%)			
After 2 h	10 (0.8%)	30 (12.3%)	47 (10.1%)			
After more than 2 h	45 (3.5%)	64 (26.2%)	172 (37.0%)			
Formula feeding of infants						
Present	664 (52.1%)	171 (67.6%)	314 (65.3%)	37.420		<0.001
Absent	610 (47.9%)	82 (32.4%)	167 (34.7%)			
Pacifier use						
Present	658 (51.6%)	151 (59.7%)	290 (60.3%)	13.393		0.001
Absent	616 (48.4%)	102 (40.3%)	191 (39.7%)			

**Table 2:** Association between complementary feeding rates and the skin-to-skin contact.

	No Complementary Feeding	Complementary Feeding	Total	*χ^2^*(1)	*p*
No skin-to-skin contact	198 (35.5%)	359 (64.5%)	557 (100%)	111.906	<0.001
Skin-to-skin contact	830 (62.1%)	506 (37.9%)	1336 (100%)		

**Table 3:** Differences in the (exclusive) breastfeeding duration regarding the onset of skin-to-skin contact.

	Skin-to-Skin Contact			*p*
Exclusive breastfeeding duration (months, M[SD))	<30 min	>30 min	t(1638)	0.003
	5.05 (2.16)	4.64 (2.49)	3.205	
Breastfeeding duration (months, M[SD])	<2 h	>2 h	t(1913)	0.021
	10.95 (8.42)	9.67 (8.05)	2.309	

**Table 4:** Types of delivery, skin-to-skin contact and infant feeding.

	Rooming-In	Non-Rooming-In	*χ*^2^ or t(df)	*p*
Type of delivery (n[%])				
Vaginal birth	1200 (94.8%)	66 (5.2%)	15.220(2)	<0.001
Elective caesarean section	229 (91.2%)	22 (8.8%)		
Emergency caesarean section	431 (89.8%)	49 (10.2%)		
Onset of skin-to-skin contact (n[%])				
Immediately	1031 (74.8%)	23 (2.9%)	1248.625(5)	<0.001
<10 min	173 (12.6%)	34 (5.8%)		
<30 min	98 (7.1%)	76 (12.9%)		
<1 h	52 (3.8%)	116 (19.7%)		
<2 h	14 (1.0%)	73 (12.4%)		
>2 h	10 (0.7%)	268 (45.4%)		
Complementary feeding (n[%])				
Not received	1001 (56.6%)	26 (20.8%)	60.123(1)	<0.001
Received	769 (43.4%)	99 (79.2%)		
Regularity of feeding (n[%])				
Demand	1468 (90.0%)	84 (81.6%)	7.241(1)	0.007
Scheduled	164 (10.0%)	19 (18.4%)		
Breastfeeding duration (months, M[SD])				
	10.80 (8.32)	9.63 (8.57)	−1523 (1931)	0.128

**Table 5:** Differences in the prevalence of exclusive breastfeeding regarding pacifier utilization.

Type of Feeding in the First 6 Months		No Pacifier Used	Pacifier Used	*p*
Non-exclusive breastfeeding	n (%)	227 (26.8%)	621 (73.2%)	<0.001
Exclusive breastfeeding		682 (58.8%)	478 (41.2%)	
Non-exclusive breastfeeding (months)	M (SD)	13.60 (8.82)	8.28 (7.09)	<0.001
Exclusive breastfeeding (months)		5.51 (1.86)	4.38 (2.47)	<0.001

## Data Availability

The data presented in this study are available on request from the corresponding author.
